# Health-related quality of life in children with cerebral palsy associated with congenital Zika infection

**DOI:** 10.1590/1984-0462/2023/41/2022016

**Published:** 2023-04-07

**Authors:** Fernanda Jordão Pinto Marques, Alessandra Lemos de Carvalho, Eliana Valverde Magro Borigato, Luiz Felipe Vieira de Oliveira, Lenamaris Mendes Rocha Duarte, Adriana Goncalves da Silva, Claret Luiz Dias Amarante, Laura Jácome de Melo Pereira, Elise Ferreira Tavares, Lilian Gleice de Sena da Costa, Carolina Alves Rezende Alcântara, Andrea Nakamura Salinas, Fernanda de Lourdes da Cunha Pinto, Gerliane Carvalho de Alcântara, Fabiana Utsch, Cinthia Ramos Diniz Silva, Dirlene Araujo dos Reis, Wilerson Marques Bessa, Rafaela Christine Dutra, Paloma Ventura, Tatiana Souza Oliveira

**Affiliations:** a Rede SARAH de Hospitais de Reabilitação, Rio de Janeiro, RJ, Brazil.; b Rede SARAH de Hospitais de Reabilitação, Salvador, BA, Brazil.; c Rede SARAH de Hospitais de Reabilitação, Brasília, DF, Brazil.; d Rede SARAH de Hospitais de Reabilitação, Fortaleza, CE, Brazil.; e Rede SARAH de Hospitais de Reabilitação, São Luis, MA, Brazil.; f Rede SARAH de Hospitais de Reabilitação, Belém, PA, Brazil.; g Rede SARAH de Hospitais de Reabilitação, Belo Horizonte, MG, Brazil.; h Rede SARAH de Hospitais de Reabilitação, Macapá, AP, Brazil.

**Keywords:** Zika virus infection, Cerebral palsy, Quality of life, Infecção por zika vírus, Paralisia cerebral, Qualidade de vida

## Abstract

**Objective:**

To describe the health-related quality of life (QOL) in children with cerebral palsy (CP) associated with congenital Zika infection.

**Methods:**

Cross-sectional study of a consecutive series of children, followed in a referral multicentric rehabilitation network in Brazil. We invited the caregivers to respond to the Brazilian version of the Caregiver Priorities & Child Health Index of Life with Disabilities (CPCHILD^TM^) questionnaire. Statistical analysis was performed with the Statistical Package for the Social Sciences (SPSS) 26.0™. We used absolute and relative frequencies for categorical variables and mean and standard deviation for continuous variables.

**Results:**

The sample consisted of 193 children, at mean age of 50.3±7.6 months. We observed a predominance of children with cerebral palsy (CP) with Gross Motor Function Classification System (GMFCS) level V (93.7%). Epilepsy (88.4%) was the most common comorbidity. CPCHILD^TM^ mean scores were activities of daily living (ADL)/personal care 43.2±12.6; positioning, transferring and mobility 33.7±16.5; comfort and emotions 84.4±15.2; communication and social interaction (CoSI) 48.2±24.3; health 70.9±17.1; and overall quality of life (OQOL) 72.1±17.1. Total score was 54.8±11.3.

**Conclusions:**

Among children with cerebral palsy (CP) related to congenital Zika syndrome, the quality of life (QOL) scores were very similar to other populations with cerebral palsy (CP). The activities of positioning, transferring and mobility had the greatest impact on health-related quality of life (QOL). Rehabilitation strategies and public policies should prioritize aspects related to mobility for this population.

## RESUMO

**Objetivo:**

Descrever a qualidade de vida relacionada à saúde em crianças com paralisia cerebral associada à infecção congênita pelo zika.

**Métodos:**

Estudo transversal de série consecutiva de casos de crianças acompanhadas em uma rede multicêntrica de reabilitação no Brasil. Convidamos o(s) cuidador(es) a responder à versão brasileira do *Caregiver Priorities & Child Health Index of Life with Disabilities* (CPCHILD^TM^). A análise estatística foi realizada com o pacote estatístico *Statistical Package for the Social Sciences* (SPSS) 26.0™. Para variáveis categóricas, foram utilizadas frequências absolutas e relativas e, para variáveis contínuas, média e desvio padrão.

**Resultados:**

Cento e noventa e três crianças, com idade média de 50,3±7,6 meses, foram incluídas. Observou-se predomínio de crianças com paralisia cerebral *Gross Motor Function Classification System* (GMFCS) nível V (93,7%). Epilepsia (88,4%) foi a comorbidade mais comum. As pontuações médias do CPCHILD^TM^ foram: atividades de vida diária/cuidados pessoais 43,2±12,6; posicionamento, transferência e mobilidade 33,7±16,5; conforto e emoções 84,4±15,2; comunicação e interação social 48,2±24,3; saúde 70,9±17,1; e qualidade de vida geral 72,1±17,1. A pontuação total foi 54,8±11,3.

**Conclusões:**

Entre as crianças com paralisia cerebral relacionada à síndrome do zika congênita, as atividades de posicionamento, transferência e mobilidade tiveram maior impacto na qualidade de vida relacionada à saúde, semelhantemente a outras etiologias de paralisia cerebral. As estratégias de reabilitação e as políticas públicas devem priorizar os aspectos relacionados à mobilidade dessa população.

## INTRODUCTION

Five years ago, Brazil faced an unprecedented Zika virus (ZIKV) epidemic, followed by the emergence of a new nosological category diagnosis: congenital Zika syndrome (CZS).^1^ CZS encompasses the spectrum of symptoms observed in infants who are exposed to ZIKV in the uterus, that may affect children’s overall growth, global development, and well-being over time.^2^ Due to its intense neurotropism and consequent severe brain malformations,^3^ ZIKV was responsible for the emergence of a generation of children with neurological disorders that may impose severe lifelong limitations and lifetime needs for these children and their families.^4^

The association between CZS and severe forms of cerebral palsy (CP) has been already documented.^4,5^ CP refers to a group of permanent disorders of posture and movement, causing activity limitation, attributed to a non-progressive damage to the developing fetal or infant brain.^6^ Around 30% of children with CP are severely affected and evolve with difficulties in activities of daily living (ADL), communication, mobility, and health.^7^

According to the World Health Organization (WHO), QOL can be defined as a broad ranging concept which includes, at a minimum, an ‘individual’s’ perception of their physical state, and the interpersonal relationships and social roles in their lives.^8^ Although it is a subjective and multifactorial dimension, it can be a useful proxy measure about the child’s health status, comfort, well-being, functional abilities and ease of caregiving.^9^ Aside from clinical and epidemiological data, its description may contribute to advocate for public health professional’s assistance and government policies. The Caregiver Priorities and Child Health Index of Life with Disabilities (CPCHILD)^9^ is a measure on the caregivers’ perspectives about the QOL, including health status, functional limitations, and well-being of children with severe CP, that has been shown to be valid, reliable and has been translated and culturally adapted to the Brazilian Portuguese.^10^

The objective of this study was to describe the health-related QOL of children with CP associated with CZS followed in a multicentric rehabilitation network in Brazil.

## METHOD

**Figure 1.**
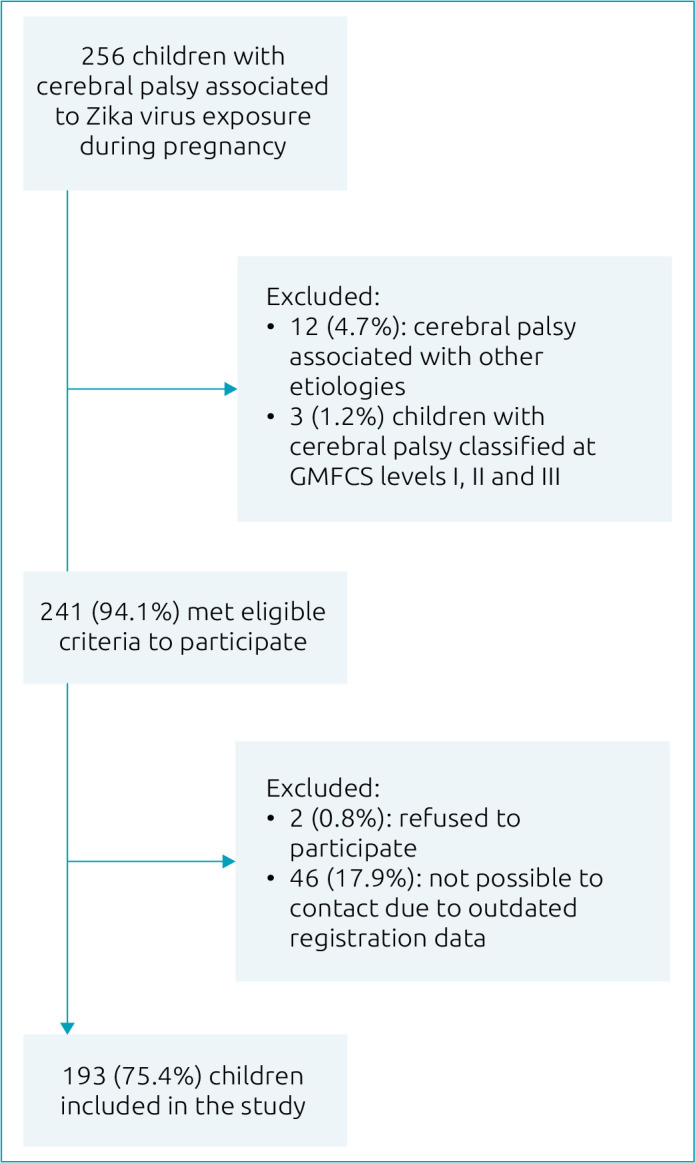
Criteria for participant selection.

This descriptive cross-sectional study comprises a consecutive series of 193 children, followed in eight units of a referral multicentric rehabilitation network across Brazil, whose mothers displayed symptoms of ZIKV infection during pregnancy (mild and self-limiting symptoms that included mild fever, maculopapular rash, headache, conjunctivitis, or myalgia^4^). The flowchart is described in Figure 1.

Eligible criteria included children with the diagnosis of PC with GMFCS levels IV or V,^11^ associated with CZS. Exclusion criteria considered other causes of microcephaly (such as hypoxic-ischemic injury, suspected genetic syndrome, and congenital cytomegalovirus [CMV] syndrome); absence of the clinical diagnosis of CP; children with CP classified at GMFCS levels I, II and III; and children without typical neuroimaging findings of CZS.

The diagnosis of CZS relied on epidemiological and clinical evaluation, neuroimaging, and laboratory data.^4^ CP diagnosis was based on the presence of motor dysfunction (essential criterion) and at least one of the other two additional criteria, i.e. abnormal neuroimaging and/or clinical history indicating risk for CP.^12^ The GMFCS comprises five levels of gross motor classification based on the child’s motor functioning, wherein level I is equivalent to minimal motor dysfunction, and level V is equivalent to significant motor impairment.^11^

Magnetic resonance imaging (MRI) with 3.0 or 1.5 Tesla resolution (GE Signa^R^ HDXT) and/or computed tomography (CT) scan (Aquilion lightning 80^R^) were performed in spontaneous sleep state. Neuroimaging findings related to ZIKV neuropathogenesis encompasses the presence of brain calcifications at the cortical/subcortical brain white matter, cortical malformations, hypoplasia/hypogenesis of the corpus callosum, and myelination delay.^13^

Clinical assessment was undertaken by a multidisciplinary team that included developmental pediatricians, physical therapists, psychologists, speech and language therapists and nurses. Plan of care, including frequency of follow up visits, varied based on each child’s individual needs.

To assess the QOL, the Brazilian version of the CPCHILD^TM10^ questionnaire was applied to the caregivers. It is an instrument that assesses the caregivers’ perspective on health status, comfort, well-being, functional skills, and ease of care in children with CP classified at GMFCS levels IV and V. The CPCHILD™ currently consists of 37 items distributed among six sections representing the following domains: ADL/personal care (nine items); positioning, transferring and mobility (eight items); comfort and emotions (nine items); communication and social interaction (seven items); health (three items); overall QOL (one item). Scores for each domain and for the total survey are standardized and range from 0 (worse) to 100 (best). There are four stages to scoring the CPCHILD^TM^: raw score per item; standardized score per item; standardized score per domain; and standardized score for total survey. Additionally, Section 7 incorporates the caregivers’ perspective on the importance of each item in the CPCHILD^TM^ questionnaire. Caregivers rated the importance of each item’s contribution to their child’s QOL on a 6-point ordinal scale anchored by 0 (least important) and 5 (most important).^9^

Statistical analysis was performed with SPSS 26.0^TM^. Descriptive and statistics were applied. For continuous variables, we used mean and standard deviation.

This study was approved by the Institutional Review Board and Research Ethics Committee (CAAE number 17050819.1.1001.0022, approved on March 13th, 2020). Prior informed consent was obtained by the parents or legal guardians.

## RESULTS

**Figure 2.**
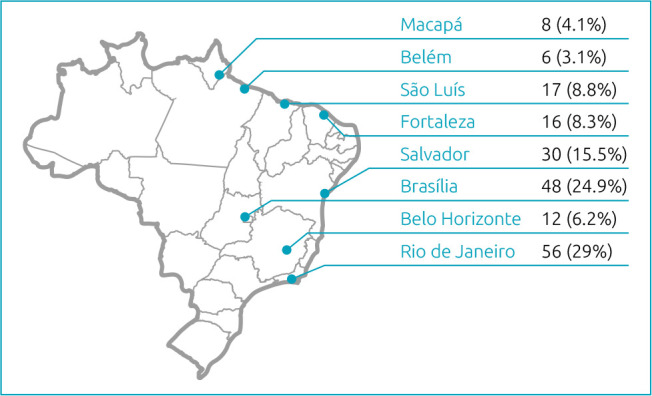
SARAH network, distributed across eight cities in Brazil, with the corresponding percentage of study participation. Children with cerebral palsy associated to congenital Zika syndrome from 18 states and the Federal District were included in the study: 72 (37.3%) from the Northeast, 72 (37.3%) from the Southeast, 31 (16.0%) from Midwest, 17 (8.8%) from the North and 1 (0.6%) from the South region of Brazil.

Figure 1 depicts the flowchart of participants selection and Figure 2 shows how the participants were distributed among the rehabilitation units. The questionnaire scores per section of the sample are summarized in Tables 1 and 2. The results of the caregivers’ perspective on the health-related of the children’s QOL are summarized for each domain on Table 3.

**Table 1.** Caregiver Priorities and Child Health Index of Life with Disabilities (CPCHILD^TM^) questionnaire scores, for sections 1 and 2, from children with cerebral palsy associated with congenital Zika virus infection, Brazil, 2020.

CPCHILD item	Standard score per item (%)
Section 1: personal care/activities of daily living (difficulty/level of assistance in the past 2 weeks)	
1. eating/drinking or being fed	46.3
2. maintaining oral hygiene	38.0
3. bathing/washing	40.2
4. toileting activities	33.9
5. changing diapers/underwear	47.2
6. putting on/taking off upper body clothing	43.6
7. putting on/taking off lower body clothing	47.5
8. putting on/wearing footwear	46.6
9. hair care	45.4
Section 2: positioning, transferring & mobility (difficulty/level of assistance in the past 2 weeks)	
10. getting in and out of bed	37.3
11. transferring into/out of a wheelchair/chair	40.8
12. sitting in a wheelchair/chair	41.3
13. standing for exercise/transfers	15.3
14. moving about in the home	33.4
15. moving about outdoors	28.8
16. getting in and out of a motor vehicle	33.1
17. visiting public places	39.6

**Table 2.** Caregiver Priorities and Child Health Index of Life with Disabilities (CPCHILD^TM^) questionnaire scores, for sections 3 to 6, from children with cerebral palsy associated with congenital Zika virus infection, Brazil, 2020.

CPCHILD item	Standard score per item (%)
Section 3: comfort & emotions (pain or discomfort in the past 2 weeks)	
18. while drinking/eating or being fed	85.9
19. during toileting	78.0
20. while dressing/undressing	89.1
21. during transfers or position changes	89.0
22. while seated	84.8
23. while lying down in bed	86.4
24. that disturbed your child’s sleep	79.5
25. how often the child was agitated, upset or angry	76.2
26. how often the child was unhappy or sad	91.4
Section 4: communication & social interaction (difficulty in each of these activities in the past 2 weeks)	
27. understanding you	59.2
28. being understood by you	62.3
29. communicating with those who don’t know your child well	31.2
30. playing alone	32.1
31. playing with others	51.5
32. attending school/childcare	46.8
33. participating in recreational activities	54.4
Section 5: health (in the past 2 weeks)	
34. how many visits to the doctor’s/hospital*	91.2
35. rate your child’s overall health	69.8
36. medications^†^	51.6
Section 6: your child’s overall quality of life	
37. rate your child’s overall quality of life	72.1

*in this item, the higher the score, the less the necessity for clinical assistance; ^†^this item refers to the number/list of medications; the higher the score, the less was the number of medications daily administered by the caregivers.

**Table 3.** Caregiver Priorities and Child Health Index of Life with Disabilities (CP-CHILD^TM^) questionnaire standardized scores per domain, from children with cerebral palsy associated with congenital Zika virus infection, Brazil, 2020.

Standardized score (per domain)	Mean (%)	Standard deviation
1. Activities of daily living/personal care	43.2	12.6
2. Positioning, transferring and mobility	33.1	13.8
3. Comfort and emotions	84.4	15.2
4. Communication and social interaction	46.8	19.4
5. Health	70.9	17.1
6. Overall quality of life	72.1	17.1
Standardized score total survey	54.4	10.5

Of the total 193 eligible children who participated in this cross-sectional study, 93 (50.3%) were male. Mean head circumference at birth was 29.1cm (3 standard deviations [SDs] or more below the mean for their age and gender). The number of mothers that reported symptoms of ZIKV infection during pregnancy in the first trimester totalled 118 (61.1%). The most frequent neuroimaging findings were brain calcifications (100.0%), malformations of cortical development (88.1%), lateral ventriculomegaly (88.3%), decreased brain volume (72.5%), hypoplasia/hypogenesis of corpus callosum (66.1%) and posterior fossa abnormalities (39.6%). Of the participants, 108 (93.3%) had CP GMFCS level V, and 13 (6.7%), level IV.

From June to December 2020, 193 caregivers (100% mothers) responded to the CPCHILD^TM^ questionnaire. The mean age of these children at the moment of recruitment was 50.3±7.5 months. Among clinical comorbidities, 168 (88.4%) presented epilepsy; 113 (76.9%) hip instability; 12 (6.2%) multiple joint contractures (arthrogryposis); 30 (15.5%) were completely fed with a gastrostomy tube due to dysphagia.

**Figure 3.**
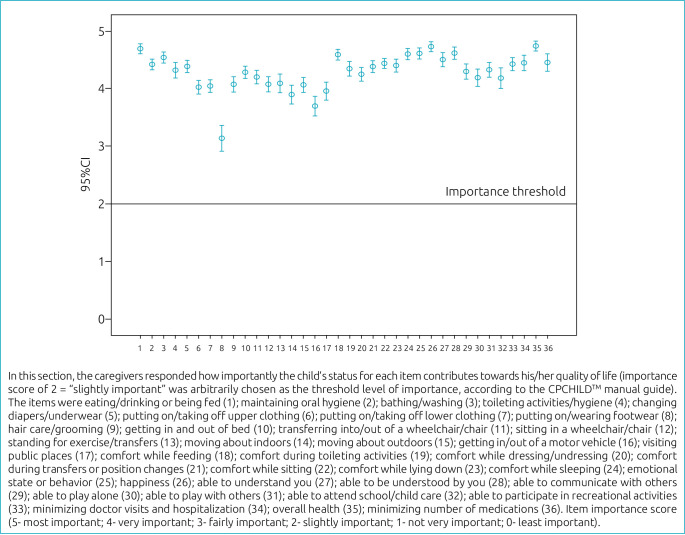
Mean relevance scores for quality of life, rated by the parents, of each item of the Caregiver Priorities and Child Health Index of Life with Disabilities (CPCHILD^TM^) questionnaire, Brazil, 2020.

Figure 3 represents how the parents rated the relevance of each item of the scale for the child’s QOL. The most relevant items reported by the parents to contribute to the child’s QOL included overall health (94.2%), happiness (94.0%), and eating/drinking or being fed (93.2%). Across the country, the mean total scores were: North 52.1±7.0, Northeast 56.4±13.4, Midwest 51.0±SD 12.5 and Southeast 55.8±8.9.

## DISCUSSION

Five years after the Zika epidemics in Brazil, families of infants born with CZS continue to struggle with daily challenges in what concerns their overall health, comfort, and QOL. As this clinical condition is associated with severe developmental disabilities,^4,5^ parents or caregivers are responsible for much of their daily needs. Caring for a child with a severe developmental disability can be challenging and have an impact on the life of the caregivers and the family.^2^ In this cross-sectional study that included 193 children with severe forms of CP associated with CZS, we aimed to evaluate their health-related QOL using the CPCHILD^TM^ questionnaire and we found that the scores were very similar to other populations of children with severe CP.^9^

Most of our sample was represented by children with the most severe clinical and functional presentation of spastic CP, which can be illustrated by the fact that more than 90% were classified as level V of the GMFCS and 15.5% required a gastrostomy tube for feeding. In this context, the activities of positioning, transferring and mobility had the greatest impact on health-related QOL. Almost 50% of the caregivers reported difficulties in maintaining the child seated in a wheelchair, in moving around outdoors, as well as attending public places, schools, and day care centers. The lack of accessibility for the physically disabled should be one of the concerns of current inclusive policies that seek to guarantee rights and adaptations in the environment, with access ramps, elevators, adaptations in bathrooms and in schools, public transport facilitators to favor the inclusion process of patients with CP.^14^

ADL/personal care also had a low score, probably related to the fact that children with CZS frequently present severe gross motor involvement (bilateral spastic quadriplegia),^4,5^ which leads to difficulties in routine activities, such as changing diapers and clothes. A possible intervention to improve caregiver´s daily challenges would be a multidisciplinary approach to the management of spasticity. Also in this section, it is noteworthy that roughly half of the caregivers considered it difficult to feed the child and this is possibly related to neurogenic oropharyngeal dysphagia that is frequent in this population.^5^ In our sample, 30 (15.5%) children were completely fed with a gastrostomy tube.

Communication and social interaction also had a low mean standardized score. This finding might be due to the high rates of severe cognitive or communication impairments not only in infants with CZS^14^ but also in children with severe forms of CP^15^ associated to other etiologies. In addition to the primary brain lesion factor, one possible explanation is that children with gross and fine motor delays are less likely capable to effectively explore the environment. Furthermore, because ZIKV is known to severely impair cortical development,^11^ it might be responsible for poor communication interaction and intellectual disability, which has been demonstrated through a high rate of children with extremely low scores in the language and cognitive domains of the Bayley Scales of Infant and Toddler development.^5^ This may hinder the ability to objectively assess comfort and QOL of these children, which further underscores the relevance of using valid and reliable proxy measures, as the CPCHILD.

The sections comfort and emotions, health and overall QOL had higher scores, possibly since all participants were accessed through rehabilitation and health services and continued to attend the follow up visits after more than five years of admission, reflecting higher levels of adherence, positive feeling, and overstating well-being. Unfortunately, this is not a reality for most of the families in Brazil, with a highly heterogeneous picture regarding access to the services required for follow-up, treatment, and rehabilitation of affected mothers and children.^16^

Section 7 of the questionnaire incorporates the caregivers’ perspective on the importance of each item in the CPCHILD^TM^. It is remarkable the high importance given by caregivers to almost all indices assessed by the questionnaire. A recent systematic review^7^ revealed that children and adolescents with CP from low-middle income countries had poor health-related QOL, especially concerning physical well-being. The interplay between disability and culture is exacerbated by poverty, where stigma and negative beliefs about disability can leave people with a disability ostracized, excluded from education, and at risk of dangerous attempts to “cure” CP.^7^ Also, factors like economic burden, uncertainty about the child’s future, and lack of provided information represent an additional strain to these caregivers.^7^

Family adaptation related to raising a child with severe, lifelong disabilities requires not only medical but also social and psychological support^2^ and alignment of the family´s expectations and rehabilitation practice. In this context, striving to understand the factors that contribute to the well-being is of great value for improving the QOL of these families.^17^ A multidisciplinary approach beyond measures of impairment and towards more holistic concepts of well-being such as health-related QOL is part of recognizing their value and fostering public health policies and a system of care that is truly centered on patients and families.

The population of children with CZS has been extensively described regarding their neurodevelopmental assessment, using instruments such as the Bayley Scales of Infant (BSID) and Toddler Development, the Gross Motor Function Measure (GMFCS), the Denver Developmental Screening Test II (DDST), the Pediatric Evaluation of Disability Inventory (PEDI), the Alberta Infant Motor Scale (AIMS), the Hammersmith Infant Neurological Examination (HINE), the Ages and Stages Questionnaire (ASQ), the Child Development Assessment Scale (CDAS) and the International Classification of Functioning, Disability and Health (ICF) framework.^18^ Nevertheless, we have not identified previous publications with a specific focus on QOL, using reliable proxy measures.

The use of parental proxy reports is particularly important in the management of severe CP, once it is a condition that compromises the child’s ability to self-report due to intellectual and/or communicative difficulties. However, proxy reports can introduce a source of systematic bias due to caregivers’ reliance on indirect cues and personal knowledge.^8^ In addition, this study was conducted during the coronavirus disease 2019 (COVID-19) pandemic, which might have influenced the caregivers’ perception about the health-related QOL of this population.

In conclusion, CZS recently was responsible for a generation of infants with severe forms of CP, classified at GMFCS levels IV and V. A child with CZS has complex needs, which represent a stark emotional strain on families. In this cross-sectional, multicentric study, the activities of positioning, transferring and mobility had the greatest impact on health-related QOL. Furthermore, the caregivers’ relevance rate on the health status, comfort, well-being, functional skills, and ease of care in children is high. Based on the modern concept of evidence-based medicine that includes the values of the patient and/or family for decision-making, it is fundamental to advocate for consecutive development of public policies and coordinated well-planned rehabilitation goals to enhance health-related QOL in the families of children with disability.
